# The Single Nucleotide Substitution T → A rs2072580 Damages the CREB1 Binding Site in the Bidirectional *SART3*/*ISCU* Promoter

**DOI:** 10.3390/genes16060713

**Published:** 2025-06-17

**Authors:** Arina Degtyareva, Elena Antontseva, Anastasia Evseenko, Konstantin Orishchenko, Tatiana Merkulova

**Affiliations:** Institute of Cytology and Genetics, Siberian Branch, Russian Academy of Sciences, Novosibirsk 630090, Russia; antontseva@bionet.nsc.ru (E.A.); evseenkoaa@bionet.nsc.ru (A.E.); keor@bionet.nsc.ru (K.O.); merkulova@bionet.nsc.ru (T.M.)

**Keywords:** regulatory SNPs, transcription factor binding sites, CREB1, EMSA, luciferase reporter assay

## Abstract

Background/Objectives: The regulatory SNPs (rSNPs) that disturb the binding of transcription factors (TFs) and alter the transcription levels of genes play a paramount role in the formation of different traits and are associated with many pathologies. The search for allele-specific events in RNA-seq and ChIP-seq data is a powerful genome-wide approach to detect rSNPs. Using this approach, we have identified the T → A rs2072580 substitution in the bidirectional *SART3*/*ISCU* promoter as a potential rSNP and demonstrated its association with colorectal cancer, relying on International Cancer Genome Consortium data. The goal of this work was to identify the TF binding site that is affected by the T → A substitution and to study the effect of this substitution on reporter gene expression in different plasmid constructs. Methods: Electrophoretic mobility shift assay (EMSA), cross-competition analysis and supershift assay, plasmid construction, and dual luciferase reporter assay. Results: The T → A rs2072580 substitution is shown to damage the binding site for ubiquitous TF CREB1 and to significantly decrease the activity of the heterologous promoter carrying the cassettes of two or three repeated CREB binding sites inserted upstream of it. However, the substitution disturbing the CREB1 binding site within the bidirectional promoter shared by *SART3* and *ISCU* inhibits the promoter activity of only the *SART3* gene but has no effect on the activity of the *ISCU* promoter. Conclusions: The performed comprehensive functional analysis of the T → A rs2072580 in the bidirectional *SART3*/*ISCU* promoter unambiguously implies it is an rSNP. These results form the background for further studies of this rSNP and its potential significance for various pathologies.

## 1. Introduction

The insight into the molecular mechanisms underlying the genetic predisposition to different diseases is necessary to understand the mechanisms of pathology development and, as a consequence, to design effective methods of their treatment and prevention [1,2]. As is known, the majority of SNPs associated with traits are confined to the noncoding genome regions, mainly the regulatory regions, such as promoters and enhancers [3,4,5]. These regulatory SNPs (rSNPs) change the structure of transcription factor binding sites (TFBSs), thereby changing the binding affinity between a TF and its cognate site and, as a result, dysregulating the gene expression and interfering with crucial biological processes [6,7,8].

Since genome-wide association studies (GWAS), the most widely used high-throughput technology when searching for the genetic variants associated with certain traits [9,10], are unable to provide the information about the functionality of detected SNPs, interest in the studies making molecular sense of the SNPs detected by GWAS at the level of both individual polymorphic sites and large-scale studies has sharply increased [6,11]. Concurrently, functional approaches to the genome-wide search for rSNPs based on either eQTL (expression quantitative trait loci) analysis [12] or allele-specific events in RNA-seq data [13], as well as ChIP-seq, DNase-seq, and ATAC-seq arrays [14,15,16,17,18], are being developed. However, a comprehensive experimental study of the found rSNPs is necessary in this case as well to gain a better understanding of the mechanism underlying various pathologies [19,20,21].

Earlier, we discovered a putative regulatory polymorphism, rs2072580 (T → A), harbored in the bidirectional promoter of *SART3* and *ISCU* genes using an integrated analysis of allele-specific events in RNA-seq and ChIP-seq data [22]. Analysis of the ICGC (International Cancer Genome Consortium) data demonstrated its association with the development of colorectal cancer (CRC) [22], further confirmed by the haplotype analysis of CRC subjects and healthy controls [23]. In addition, genotyping showed the correlation between rs2072580 and breast cancer [24].

The putative regulatory function of rs2072580 is confirmed by the results of eQTL mapping [12]. According to the GTEx Consortium atlas, rs2072580 is an eQTL for the *SART3* and *ISCU* genes in the common promoter region in which it is located, as well as for the *FICD2* and *WSCD2* genes situated at a distance of 46 and 432 kb, respectively. Note that all these genes are shown to be linked with malignancy.

In particular, the SART3 protein expression is long known to be very low in both the normal tissues and non-proliferating cells, being considerably increased in several malignant tumor cell lines and cancer tissues; this has even suggested using SART3 as a potential antigen for cancer immunotherapy [25,26,27]. An increased *SART3* gene expression was observable in the cells and tissues with different cancer phenotypes at the transcriptional level too [28,29]. Moreover, a *SART3* knockdown was shown to reduce the ability of the A549 cell line (human lung cancer) to develop xenograft tumors in nude mice [30]. A change in the *ISCU* gene expression can also be involved in carcinogenesis, thanks to an important role of this gene in iron homeostasis [31,32,33]. The *ISCU* expression was shown to be decreased in most human liver cancer tissues [32], melanomas [34], and parasympathetic paragangliomas [35]. In addition, it is known that the cancers with low *ISCU* have a worse prognosis [31], as, for example, in the case of pediatric adrenocortical tumors [36] or oropharyngeal squamous cell carcinomas [37]. *ISCU* gene knockdown enhanced the self-renewal of colon tumor-initiating cells [38]. An increased expression of *WSCD2* is associated with a favorable prognosis for glioma patients [39]. On the other hand, the breast cancer subjects are at a higher risk of mortality at a low expression of *WSCD2* combined with large nuclei [40]. *FICD* has been identified as a candidate radioresistance gene in CRC cell lines [41].

In this study, we aimed to find out whether the rs2072580 T → A substitution influences the binding of any transcription factor, to identify the corresponding transcription factor, and to assess the effect of this substitution on the reporter gene expression in different plasmid constructs.

## 2. Materials and Methods

### 2.1. Oligonucleotides

All oligonucleotides used in this study—for genotyping (Table S1), EMSA (Table S2), plasmid construction (Tables S3 and S4), and plasmid construct verification (Table S5)—were synthesized by Biosset (Novosibirsk, Russia).

### 2.2. Cell Cultures

HepG2, MCF-7, and Caco-2 cells were cultivated in the DMEM/F12 medium supplemented with L-glutamine (BIOLOT, St Petersburg, Russia), 10% fetal bovine serum (Thermo Scientific HYCLONE, Logan, UT, USA), or 20% fetal bovine serum (for Caco-2 cells). Growth medium contained 100 units/mL penicillin and 100 mg/mL streptomycin (BIOLOT, Russia); growth conditions, 5% CO_2_ at 37 °C.

### 2.3. Genomic DNA Isolation and Genotyping

Genomic DNA was extracted from the buccal epithelium of six healthy donors using a standard phenol/chloroform technique with ethanol precipitation. The study was approved by the Ethical Committee of the Institute of Molecular Biology and Biophysics, Siberian Branch, Russian Academy of Medical Sciences; informed consent was obtained from all subjects. The region containing the target SNP (rs2072580) was amplified with PCR (see Table S1 for the primer pair). The primers were designed using the Primer-BLAST tool (https://www.ncbi.nlm.nih.gov/tools/primer-blast/ (accessed on 12 April 2018)). The genotyping for rs2072580 was carried out by Sanger sequencing using Applied Biosystems BigDye v3.1 sequencing chemistry at the Genomics Core Facility with the Institute of Chemical Biology and Fundamental Medicine, Siberian Branch, Russian Academy of Sciences, Novosibirsk, Russia. Homozygous samples were used for plasmid construction.

### 2.4. Preparation of Nuclear Extracts and Electrophoretic Mobility Shift Assay (EMSA)

The preparation of nuclear extracts and Electrophoretic Mobility Shift Assays (EMSAs) has been described previously [42]. Anti-CREB1 antibodies (AF3189, Affinity Biosciences, Beijing, China) were used.

### 2.5. Plasmid Construction, Transfection, and Luciferase Reporter Assay

Plasmid constructs containing single (31 bp), double (62 bp), and triple (93 bp) inserts with the A/T rs2072580 site were created. The synthesized oligonucleotides with sticky ends corresponding to *XhoI* and *HindIII* restriction sites (Table S3) were annealed and ligated into pGL4.23 [minP/luc] (Promega, Madison, WI, USA). Two fragments containing *SART3* and *ISCU* common promoter region harboring rs2072580, first, −493 to +31 with respect to the transcription initiation site (TSS) of *SART3,* and second, –321 to +203 with respect to the TSS of *ISCU* (Figure S1), were amplified from genomic DNA. The forward primer with the *HindIII* restriction site and reverse primer with the *XhoI* restriction site at the 5′ ends were used for PCR of the *SART3*-oriented bidirectional promoter. Vice versa, the forward primer with the *XhoI* restriction site and reverse primer with the *HindIII* restriction site at the 5′ ends were used for PCR of the *ISCU*-oriented bidirectional promoter (Table S4). The amplification with the insert-specific primers ensured the correct orientation of *SART3* and *ISCU* promoters. The PCR products were digested with *XhoI* and *HindIII* (SibEnzyme, Novosibirsk, Russia) and ligated into the pGL3-basic (Promega) using T4 DNA ligase (SibEnzyme) to construct recombinant plasmids pGL3-ISCU-A, pGL3-ISCU-T, pGL3-SART3-A, and pGL3-SART3-T. Plasmid DNA was extracted and purified with the Plasmid Midiprep 2.0 Kit (Evrogen, Moscow, Russia) and verified by Sanger sequencing with the corresponding primers (Table S5).

Approximately 5 × 10^4^ HepG2/MCF-7 cells were plated onto a 24-well plate in antibiotic-free media 24 h prior to transfection. Then, the cells were cotransfected with recombinant plasmids and pRL-TK (Promega) using TransIntro transfection reagent (TransGen Biotech, Beijing, China) according to the manufacturer’s protocol. The cells were incubated with the transfection reagent for 10 h at 37 °C and replated in fresh medium. After 24 h, both firefly and Renilla luciferase activities were measured using the Dual Luciferase Assay kit (Promega) according to the manufacturer’s protocol.

### 2.6. Statistical Analysis

To compare the relative luciferase activity of the reporter constructs containing minor and major alleles, statistical analysis was performed using Student’s *t*-test and R Statistical Software [R Core Team (2021) R: A Language and Environment for Statistical Computing. R Foundation for Statistical Computing, Vienna (https://www.R-project.org (accessed on 10 October 2023))]. All experiments were repeated at least three times, and each included three replicates. In the statistical analyses, *p* < 0.05 was considered statistically significant.

## 3. Results

### 3.1. Single Nucleotide Substitution T → A rs2072580 Disrupts CREB1 Transcription Factor Binding Site

To assess the effect of T → A rs2072580 substitution on the binding of any TF, we analyzed the retardation of the corresponding DNA probes (Table S2) in gel by nuclear extract proteins (EMSA). Three cell lines were used for this purpose: human hepatocarcinoma cells HepG2, human breast cancer cells MCF7, and human colorectal adenocarcinoma cells Caco-2.

**Figure 1:**
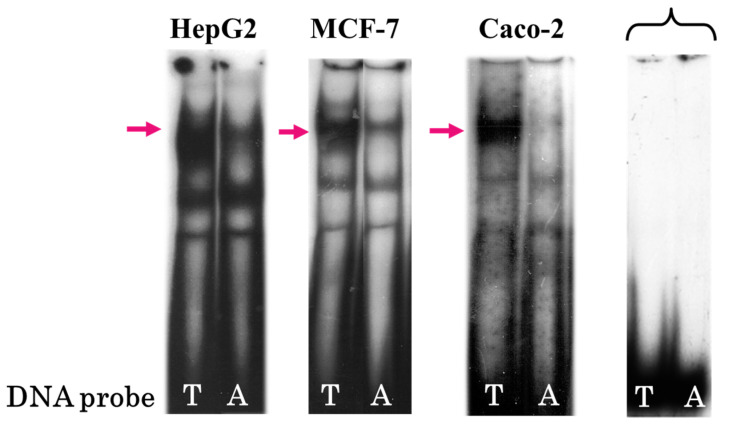
Electrophoretic mobility shift assays (EMSAs) with the DNA probes containing either rs2072580-T (**left**) or rs2072580-A (**right**) using HepG2, MCF-7, and Caco-2 nuclear extracts; brace denotes free DNA probes and arrows, the DNA–protein complex is prevalently formed in the case of allele T.

As is evident from Figure 1, the T → A rs2072580 substitution leads to a weakening of the upper band, observable in all three cell lines and suggesting a disturbance of the binding site for a ubiquitous TF.

**Figure 2:**
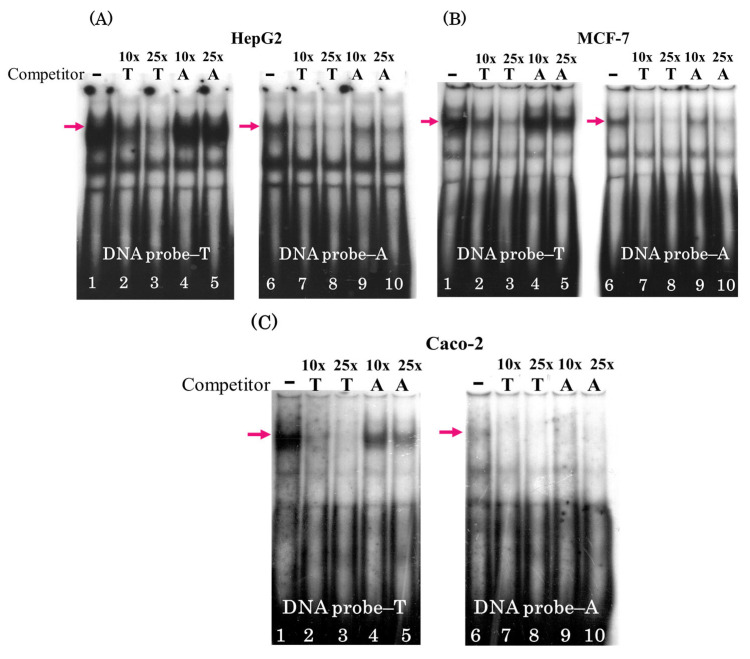
Cross-competition analysis. Gel shift assays were performed with the DNA probes containing either rs2072580-T or rs2072580-A using (**A**) HepG2, (**B**) MCF-7, and (**C**) Caco-2 nuclear extracts: lanes 1–5, labeled DNA probe carrying allele T; lanes 6–10, labeled DNA probe carrying allele A; lanes 1 and 6, without competitor; and lanes 2–5 and 7–10, 10- and 25-fold excess of unlabeled oligonucleotide; arrow denotes the specific protein complex bound by the T allele.

Cross-competition analysis using the same cell lines confirmed the preferential binding of this TF to the DNA probe carrying the T allele. Figure 2 shows a considerably stronger weakening of the upper band with increasing excess of a cold competitor carrying allele T: compare lanes 2, 3 and 7, 8 (Figure 2A–C) and lanes 4, 5 and 9, 10, when the excess of unlabeled oligonucleotide carrying allele A is used as a competitor.

The MotifbreakR (2.2) software package [43] was used to predict the TFBSs affected by T → A rs2072580. This allowed us to select the candidate TFs destroyed by the T → A substitution, namely CREB1, PAX3, and AP1. As for the FOXK1 TF, this substitution improved the site (Table 1).

**Figure 3:**
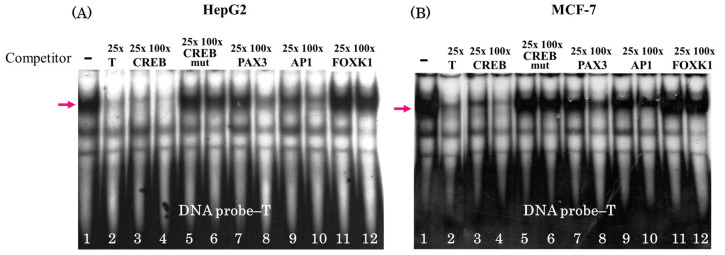
Competition analysis in EMSA experiments using (**A**) HepG2 and (**B**) MCF-7 nuclear extracts. Lane 1, without competitor; lane 2, 25-fold excess of cold oligonucleotide containing T allele; and lanes 3–12, 25-fold and 100-fold excess of competitor oligonucleotides corresponding to TF binding sites; arrow denotes specific protein complex bound by the T allele of rs2072580.

The unlabeled oligonucleotides carrying the binding sites for these TFs (Table S2) were used in competitive analysis (Figure 3). The oligonucleotide corresponding to the CREB binding site emerged as the strongest competitor for complex formation (Figure 3A,B, lanes 3 and 4). In addition, the absence of competition when adding the oligonucleotide with a mutated site is another strong argument favoring CREB binding (Table S2; Figure 3A,B, see lanes 5 and 6 for CREB mut). Moreover, the oligonucleotides carrying PAX3 and AP1 binding sites appeared as weak competitors in 100-fold excess amounts (Figure 3, lanes 8 and 10) most likely as a result of the similarity of the motifs to that of CREB, while the sequence corresponding to the FOXK1 binding site does not contain a similar motif and does not compete even in a large excess (Figure 3A,B, lanes 11 and 12).

**Figure 4:**
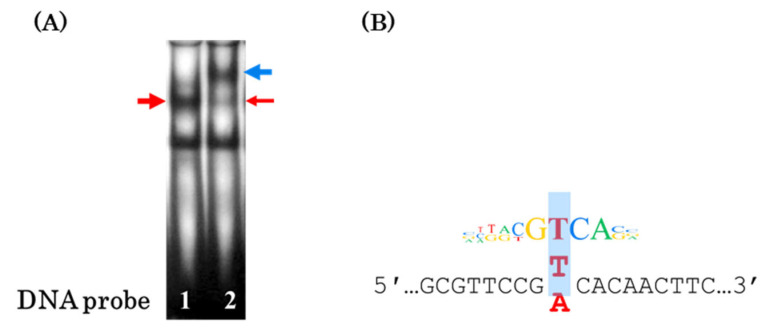
Substitution T → A destroys the CREB1 binding site. (**A**) EMSA was conducted using anti-CREB1 antibodies. Red arrows denote the DNA probe–CREB1 complex and blue denotes supershift in the presence of antibodies. (**B**) CREB1 motif logo from the MotifbreakR aligned to the sequence of the identified CREB1 binding site; blue area highlights rs2072580 T/A.

Thus, CREB1 is the most likely TF, the binding site of which is destroyed by the T → A substitution (Figure 4B). The EMSA experiments with the antibodies against CREB1 completely confirmed this assumption. Addition of these antibodies almost completely eliminated the band corresponding to the DNA probe–protein complex, while the supershift (DNA probe–protein–antibody complex) appeared (Figure 4A, blue arrow).

### 3.2. The Oligonucleotide T → A rs2072580 Substitution Destroys the CREB1 Binding Site and Decreases the Activity of the Corresponding Regulatory Element

**Figure 5:**
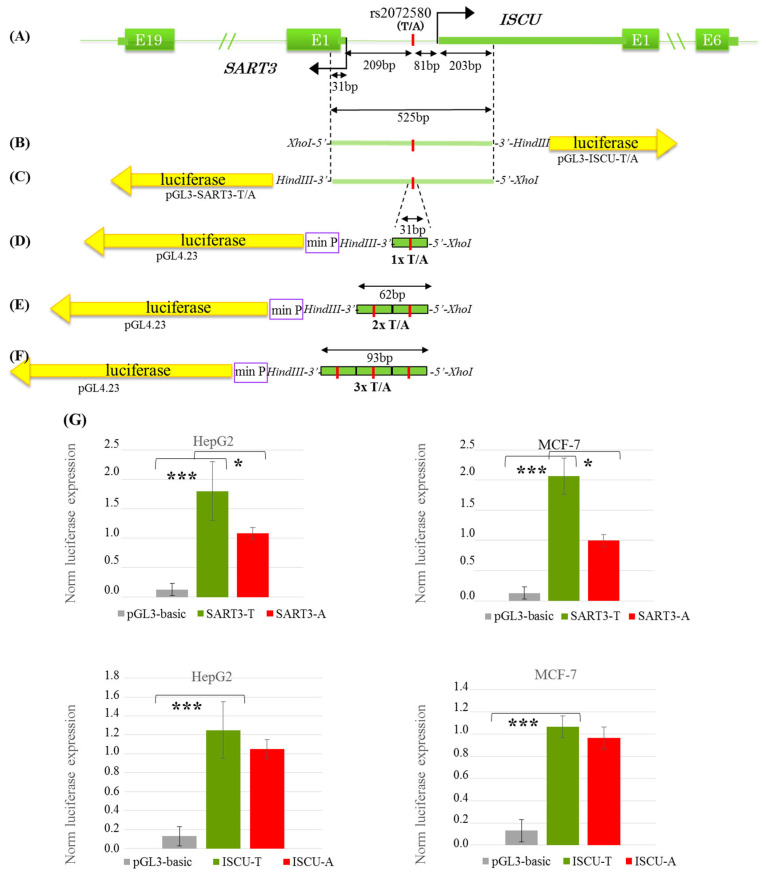
The effect of the T → A rs2072580 substitution on the activities of *SART3* and *ISCU* promoters. (**A**) Scheme of bidirectional promoter region shared by *SART3* (NM_001410983.1, NCBI RefSeq genes) and ISCU (NM_001301141.1, NCBI RefSeq genes); angle arrows denote the transcription start sites (TSSs) and green rectangles denote exons (**B**–**F**). Schemes of the recombinant plasmids used in dual luciferase assays: bidirectional promoter constructs with (**B**) forward or (**C**) inverted orientations; constructs with (**D**) single, (**E**) double, and (**F**) triple oligonucleotide inserts. (**G**) Relative luciferase activity in the HepG2 and MCF-7 cells transfected with the constructs containing *ISCU*- and *SART3*-oriented promoter regions harboring alternative rs2072580 alleles (green column, allele T and red column, allele A); and the gray column shows the empty pGL3-basic vector. The data of three independent experiments are shown as mean values ± SD, * *p* < 0.05, significant difference between alleles; and *** *p* < 0.001, significant difference between the promoter-containing constructs and empty vector (Student’s *t*-test).

**Figure 6:**
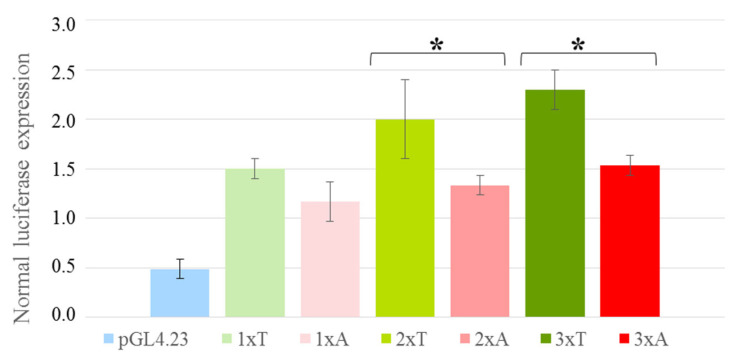
The effect of rs2072580 in the constructs containing single, double, and triple inserts of the CREB1 binding site when transfected into HepG2 cells: green columns show the T allele; red columns show the A allele; color intensity reflects the increasing number of inserts; and the blue column shows the empty pGL4.23 vector. All data were normalized to Renilla luciferase internal reference. The data from three independent experiments are shown as mean values ± SD; * *p* < 0.05, significant difference between the alleles (Student’s *t*-test).

The oligonucleotides reproducing EMSA DNA probes A and T (except for the attached sticky ends; Table S3) were used to assess the effect of T → A in the CREB1 binding site on the activity of the corresponding regulatory element. These oligonucleotides were inserted directly upstream of the pGL4.23 minimal promoter as either single elements or the cassettes of two or three repeated TFBSs (Figure 5D–F). As is evident from Figure 6, the T → A substitution in the case of a single insert causes a weak decrease in the reporter gene expression (the difference is statistically insignificant) versus a distinct statistically significant decrease (by 35%) in the reporter gene expression for allele A observable when using the cassettes of two or three repeated TFBSs.

### 3.3. The T → A rs2072580 Substitution Destroys the CREB1 Binding Site abd Decreases the Activity of SART3 Promoter but Has No Effect on the Activity of the ISCU Promoter Within the Bidirectional SART3/ISCU Promoter

The rs2072580 substitution is harbored in the bidirectional promoter of *SART3* and *ISCU* genes (Figure 5A and Figure S1). Correspondingly, four constructs were created using the no-promoter plasmid pGL3-basic carrying the promoter region in the *ISCU* orientation with alternative T → A rs2072580 alleles (forward orientation, Figure 5B) and the same constructs with the promoter region in the *SART3* (inverted orientation, Figure 5C). HepG2 (hepatocarcinoma) and MCF-7 (breast cancer) cell lines were used for transfection. The constructs with both orientations had a significantly higher level of promoter activity as compared with the no-promoter control in both cell lines (Figure 5G). However, the effect of T → A substitution on the reporter gene expression was observed in both cell lines only in the constructs with the *SART3* orientation (Figure 5G). The T → A substitution in the case of HepG2 cells reduces the expression of the reporter gene by 39% and MCF7 by 52%. Note that the disruption of the CREB1 binding site within the promoter region decreased the reporter gene activity, similar to what we observed within the isolated regulatory element (Figure 6).

## 4. Discussion

Recently, the research into the regulatory functions of individual SNPs has become ever more relevant [6,7,44]. This research mainly focuses on the polymorphic sites detected by GWAS. A particular combination of the methods of a vast methodological toolkit is used for this purpose to determine the effect of a nucleotide substitution on the binding of certain or several TFs, to identify this/these TF(s), to study the effect of the nucleotide substitution on in vitro and in vivo transcription, and so on [6,11,44]. Different combinations of the methods from the same toolkit are also used to study potential regulatory SNPs (rSNPs) discovered using genome-wide functional approaches (eQTL analysis and search for allele-specific events in RNA-seq, ChIP-seq, DNase-seq, and ATAC-seq data) [19,20,21].

Initially, we identified T → A rs2072580 as a potential regulatory polymorphism utilizing one of the functional approaches, namely, integrated analysis of allele-specific events in RNA-seq and ChIP-seq data [22]. The frequency of the rs2072580 minor allele T (MAF) varies in different ethnic groups from 0.09 (East Asian populations, gnomAD genomes v4.1 [https://gnomad.broadinstitute.org/ (accessed on 10 February 2025)]) to 0.42 (European populations, 1000 Genomes Project Phase 3 [https://www.internationalgenome.org/home (accessed on 10 February 2025)]). Our data on a putative regulatory function of rs2072580 are confirmed by the results of eQTL analysis, demonstrating the effect of the allele in the expression level of four genes: *SART3* and *ISCU*, harboring this polymorphic site in their common promoter region, and *WSCD2* and *FICD2*, situated as a distance of 46 and 432 kb, respectively [12].

In this study, we used EMSA with nuclear extracts of human cell lines of different origins (HepG2, hepatocarcinoma; MCF7, breast cancer; and Caco-2, colorectal adenocarcinoma) to demonstrate that the T → A rs2072580 substitution destroyed the binding site of a certain (putatively ubiquitous) TF (Figure 1 and Figure 2). The experiments on the competition of labeled DNA probe and cold oligonucleotides that corresponded to the predicted TFBSs suggested CREB1 as the most likely candidate TF, the binding site of which was disrupted by the studied nucleotide substitution (Figure 3). The use of specific antibodies completely confirmed this assumption (Figure 4).

CREB1 (cAMP response element-binding protein 1) is a ubiquitously expressed nuclear transcription factor of the CREB/ATF family; it has a conserved basic region/leucine zipper (bZIP) domain [45,46]. CREB1 is involved in the control of numerous processes, including cell growth, differentiation, survival, apoptosis, and metabolism in a cell-type-specific manner, implemented via the up- and downregulation of many target genes [46,47,48,49,50]. The diversity of CREB1 effects is provided through a multitude of mechanisms, such as the formation of spliced CREB isoforms functioning as transcriptional activators or repressors; modulation of CREB activity by differential and combinatorial phosphorylation; and its interaction with other TFs and distinct transcriptional coactivators [46,48].

We used a luciferase reporter assay to demonstrate that the T → A substitution destroying the CREB1 binding site decreases the transcriptional activity of the corresponding regulatory element (Figure 6). In this experiment, we used the long-known and effective practice of inserting a cassette of two–three repeated TFBSs upstream of the heterologous promoter, which allows a more pronounced regulatory effect to be recorded [51,52,53,54].

The CREB1 binding site that we identified is harbored by a bidirectional promoter shared by the *SART3* and *ISCU* genes. As is known, the transcription at bidirectional promoters is initiated from separate core promoters and completed by the formation of two full-length stable transcripts [55]. It is also known that a larger part of the bidirectional gene pairs utilizes the same set of TFs (and, correspondingly, their binding sites within the bidirectional promoter) for a coordinated transcription of both genes, which, as a rule, are functionally related [55,56,57]. However, the sets of TFs regulating the activities of different core promoters differ to a certain degree in some variants of bidirectional promoters [57]. Our data suggest that the bidirectional *SART3/ISCU* promoter belongs to the second group because a decrease in the transcriptional activity in the case of the T → A substitution was observable only when the reporter gene was under the control of the *SART3* gene promoter. If the reporter gene was under the control of the *ISCU* gene promoter, the substitution had no effect on its expression (Figure 5G). In part, this asymmetry can be explained by differences in the location of the predicted TFBSs in this region relative to both the experimentally confirmed CREB1 binding site and the transcription start sites (Figure S2). The absence of any data on the functional link between the protein products of *SART3* and *ISCU* genes confirms that the assumption that these genes have different control modes is logical. It is known that SART3 (also known as Tip110) is an RNA-binding protein with a critical role in the pre-mRNA splicing [28,58,59], while ISCU is a component of the iron-sulfur (Fe-S) cluster scaffold involved in the function of a set of various enzymes, in particular, regulating metabolism, iron homeostasis, and the oxidative stress response [60,61].

The SART3 gene expression is elevated in the overwhelming majority of malignant neoplasms as compared with the initial tissues [28,62], suggesting its promoting role in carcinogenesis. However, SART3 has also been shown to act as a tumor suppressor [63]. Therefore, it is difficult to make a definitive conclusion about the role of T → A substitution (rs2072580) in malignancy. Moreover, the studied bidirectional promoter is situated within a larger regulatory element, Promoter/Enhancer GH12J108559 (chr12:108559380-108571769; 12.4 kb), which interacts with genes *FICD*, *UBE3B*, *PWP1*, *PRDM4, HSALNG0143722*, and *TMEM119* and can control their expression according to the GeneHancer database [64]; consequently, this suggests a putative regulatory effect of rs2072580 on these genes. An association with cancer has been shown for almost all of these genes, namely, *FICD* gene is associated with CRC [41]; *UBE3B*, with breast cancer [65]; *PWP1*, with non-small cell lung cancer [66], gastric cancer [67], and hepatocellular carcinoma [68]; *PRDM4*, with gastric cancer [69], prostate cancer [70], and cervical carcinoma [71]; and *TMEM119*, with osteosarcoma [72], ovarian cancer [73], breast cancer [74], and non-small cell lung cancer [75]. Thus, the general picture that emerges is much more complex and, thus, requires further studies.

## Figures and Tables

**Table 1:** TFs, the binding sites of which are affected by T → A rs2072580, detected with the help of the MotifbreakR software package.

REF	ALT	scoreRef	scoreAlt	alleleDiff	Effect	TF
T	A	12.639493	14.623237	1.9837437	Strong	FOXK1
T	A	9.203210	7.564058	−1.6391518	Strong	CREB1
T	A	9.069532	7.481721	−1.5878104	Strong	PAX3
T	A	5.061990	4.043738	−1.0182518	Strong	AP1 (FOSL1:JUND)

REF is reference allele; ALT, alternative allele; and TF, transcription factor.

## Data Availability

The original contributions presented in the study are included in the article, further inquiries can be directed to the corresponding author.

## Supplementary Materials

The following supporting information can be downloaded at https://www.mdpi.com/article/10.3390/genes16060713/s1, Figure S1: UCSC Genome Browser visualization (GRCh38/hg38) of the common promoter region of *SART3* and *ISCU* genes, and SNP rs2072580; Figure S2: An in silico analysis of the bidirectional *SART3*/*ISCU* promoter; Table S1: Primers for rs2072580 genotyping; Table S2: Oligonucleotides for EMSA; Table S3: Oligonucleotides with sticky ends corresponding to *XhoI* and *HindIII* restriction sites for single, double, and triple inserts containing the A/T rs2072580 site; Table S4: Primers for *SART3*/*ISCU* promoter region amplification; Table S5: Primers for plasmid DNA verification by Sanger sequencing.
