# Supplementary material for: The Single Nucleotide Substitution T → A rs2072580 Damages the CREB1 Binding Site in the Bidirectional SART3/ISCU Promoter
Source: Genes (Basel). 2025 Jun 17;16(6):713. doi: 10.3390/genes16060713 (PMC12192476; doi:10.3390/genes16060713)
Supplement: Supplementary file 1 [file genes-16-00713-s001.zip › genes-3688972-supplementary.pdf]

Supplementary Materials

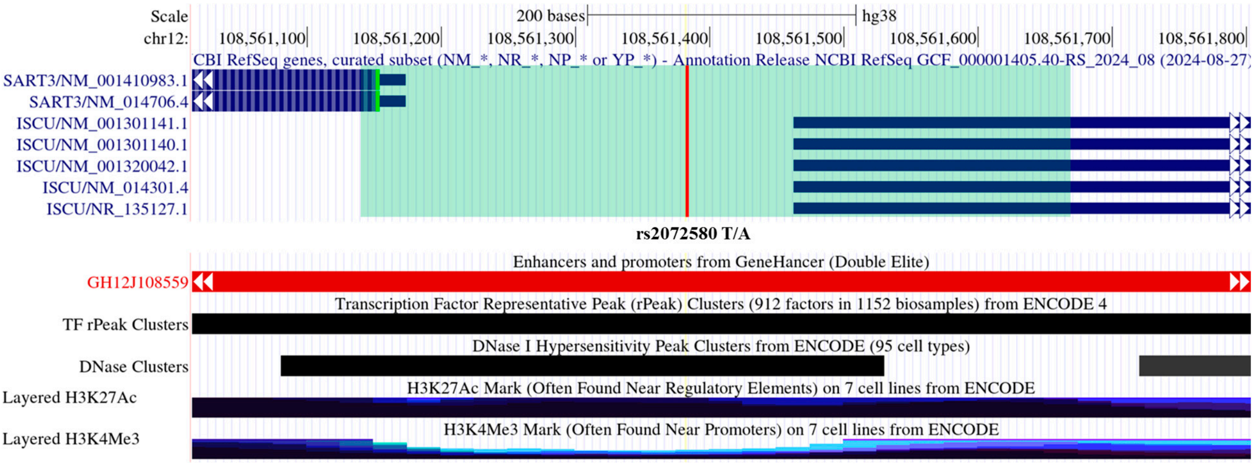

Figure S1. UCSC Genome Browser visualization (GRCh38/hg38) of the common promoter region of *SART3* and *ISCU* genes, and SNP rs2072580. NCBI RefSeq data for *SART3* and *ISCU* are presented (dark blue transcripts). The light green area highlights the *SART3* and *USCU* promoter region, the vertical red line – location of the rs2072580. Red track denotes a Promoter/Enhancer element – GH12J108559, black tracks – transcription factor representative peak clusters and DNase I hypersensitivity peak clusters, H3K27Ac and H3K4me3 marks

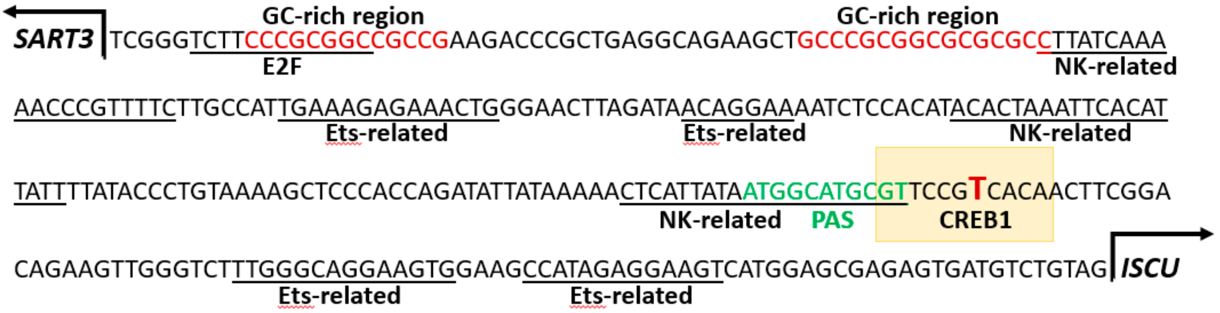

Figure S2. An *in silico* analysis of the bidirectional *SART3/ISCU* promoter. An *in silico* analysis of the 291 bp DNA sequence of the bidirectional *SART3/ISCU* promoter was conducted using motifs from the HOCOMOCO database (https://hocomoco11.autosome.org/). The experimentally confirmed CREB1 binding site binding site disrupted by the T→A substitution is highlighted in yellow square. GC-rich regions are marked in red. The putative binding sites for families of transcription factors are underlined or printed in green when superimposed on each other. The labeling of motifs are indicated below the line.

Table S1. Primers for rs2072580 genotyping

Oligonucleotide name	Sequences (5′→3′)
rs2072580_F	CCGCAGTCGCCATCTTGC
rs2072580_R	ACATAAAGGGGAGGAATCTGGG

Table S2. Oligonucleotides for EMSA

Oligonucleotide name	Sequences (5′→3′)
DNA-probes oligonucleotides containing A/T allele rs2072580 site	

SART3_T	ATGGCATGCGTTCCG*T*CACAACTTCGGACAG
SART3_A	ATGGCATGCGTTCCG*A*CACAACTTCGGACAG
Competitor oligonucleotides	
CREB	AGAGATTGCCTGACGTCAGAGAGCTAG
CREB_mut	AGAGATTGCCTG**TG**GTCAGAGAGCTAG
PAX3	GATTTCTTCCAATTAGTCACGCTTGAGTG
AP1	CGCTTGATGACTCAGCCGGAA
FOXK1	CCTGTCTTTGCTCCTTGTTTGGGAAGCGAGTGGG

Competitor oligonucleotides were taken from the Gel Shift Oligonucleotides catalog (Santa Cruz Biotechnology, Inc., USA). CREB motif mutant nucleotides are marked in bold style.

Table S3. Oligonucleotides with sticky ends corresponding to *XhoI* and *HindIII* restriction sites for single, double and triple inserts containing A/T rs2072580 site

Oligonucleotide name	Sequences (5′→3′)
SART3_1A-*HindIII*_F	agcttATGGCATGCGTTCCG**A**CACAACTTCGGACAG
SART3_1A-*XhoI*_R	tcgagCTGTCCGAAGTTGTG**T**CGGAACGCATGCCAT
SART3_2A-*HindIII*_F	agcttATGGCATGCGTTCCG**A**CACAACTTCGGACAGATGGCATGCGTTCCG**A**CACAACTTCGGACAG
SART3_2A-*XhoI*_R	tcgagCTGTCCGAAGTTGTG**T**CGGAACGCATGCCATCTGTCCGAAGTTGTG**T**CGGAACGCATGCCAT
SART3_3A-*HindIII*_F	agcttATGGCATGCGTTCCG**A**CACAACTTCGGACAGATGGCATGCGTTCCG**A**CACAACTTCGGACAGATGGCATGCGTTCCG**A**CACAACTTCGGACAG
SART3_3A-*XhoI*_R	tcgagCTGTCCGAAGTTGTG**T**CGGAACGCATGCCATCTGTCCGAAGTTGTG**T**CGGAACGCATGCCATCTGTCCGAAGTTGTG**T**CGGAACGCATGCCAT
SART3_1T-*HindIII*_F	agcttATGGCATGCGTTCCG**T**CACAACTTCGGACAG
SART3_1T-*XhoI*_R	tcgagCTGTCCGAAGTTGTG**A**CGGAACGCATGCCAT
SART3_2T-*HindIII*_F	agcttATGGCATGCGTTCCG**T**CACAACTTCGGACAGATGGCATGCGTTCCG**T**CACAACTTCGGACAG
SART3_2T-*XhoI*_R	tcgagCTGTCCGAAGTTGTG**A**CGGAACGCATGCCATCTGTCCGAAGTTGTG**A**CGGAACGCATGCCAT
SART3_3T-*HindIII*_F	agcttATGGCATGCGTTCCG**T**CACAACTTCGGACAGATGGCATGCGTTCCG**T**CACAACTTCGGACAGATGGCATGCGTTCCG**T**CACAACTTCGGACAG
SART3_3T-*XhoI*_R	tcgagCTGTCCGAAGTTGTG**A**CGGAACGCATGCCATCTGTCCGAAGTTGTG**A**CGGAACGCATGCCATCTGTCCGAAGTTGTG**A**CGGAACGCATGCCAT

SNP position is marked in bold style; sticky ends are indicated in lowercase letters.

Table S4. Primers for *SART3/ISCU* promoter region amplification

Oligonucleotide name	Sequences (5′→3′)
SART3- HindIII _F	NNNaagcttCCGCAGTCGCCATCTTGC
SART3- XhoI _R	NNNctcgaGACATAAAGGGGAGGAATCTGGG
ISCU- XhoI _F	NNNctcgagCCGCAGTCGCCATCTTGC
ISCU- HindIII _R	NNNaagcttGACATAAAGGGGAGGAATCTGGG

XhoI and HindIII restriction sites are indicated in lowercase letters.

Table S5. Primers for plasmid DNA verification by Sanger sequencing

Oligonucleotide name	Sequences (5′→3′)
pGl3-Basic_F	CTAGCAAAATAGGCTGTCCC
pGl3-Basic_R	TCTTCCAGCGGATAGAATGG

pGL4.23_F	TAGCAAAATAGGCTGTCCC
pGL4.23_R	CGCTTCATGGCTTTGTG
